# Essays on the Subcutaneous Method of Operating, Preceded by an Historical Introduction on Its Origin and Nature

**Published:** 1842-07

**Authors:** 


ART. XI.

*Essais sur la Méthode Sous-cutanée, etc., précédés d'une Introduction Historique sur l'Origine et la Constitution de cette Méthode.* Par le Dr. JULES GUERIN.—*Paris*, 1841. 8vo, pp. 126.

*Essays on the Subcutaneous Method of Operating, preceded by an Historical Introduction on its Origin and Nature.* By Dr. J. GUERIN. —*Paris*, 1841.

THIS is a pamphlet designed to claim for the author the exclusive merit of demonstrating that subcutaneous incisions, made with exclusion of air, do not inflame or suppurate, but immediately cicatrize ; and to establish thereon a general principle applicable to a variety of chirurgical operations.

The author sets out by the admission that subcutaneous section of the tendo Achillis was the first impulse towards the formation of the system, and professes to trace by whom and in what manner this operation, "a mere *empirical fact*" attained the practical development it had received when he commenced his investigations.

"The first operators had divided at one stroke the tendon and skin by a transverse incision. The first step towards the subcutaneous method and one which resulted from blind instinct, was the division of the sterno-mastoid upon a director by means of a longitudinal incision in the integuments parallel to and above the muscular fibres ; the intention having been to diminish the extent of the inflammation by lessening the size of the wound and to avoid an ugly cicatrix. [We cannot conceive by what justice a modification of an operation founded on such cogent reasons can be attributed to blind instinct.—*Rev.*] Delpech advanced another step towards the method: he proposed, and was the first to execute the subcutaneous section of the tendo Achillis...........by dividing it in situ beneath the integuments through an incision parallel to the tendon, but open on each side. Delpech sought to prevent exfoliation of the tendon by not exposing it.—[In this he was not successful, as suppuration and exfoliation of the tendon retarded the cure many months.—*Rev.*] Subsequently, in 1822, Dupuytren divided one portion of the sterno-mastoïd beneath the integuments, preferring this method, (the patient being a young girl,) in order to avoid disfigurement. He made a smaller incision than Delpech did in the case of the tendo Achillis. This celebrated surgeon therefore effected a novel and practical improvement in the operation, but does not appear to have suspected the physiological import of the result he obtained. Stromeyer adopted in 1833 section of the tendo Achillis after the idea of Delpech: [This statement is incorrect as regards the date of Stromeyer's first operation, which took place in 1831, see Stromeyer's Beiträge, p. 60.—*Rev.*] he diminished the size of the openings in the integuments so as to fulfil the indications described by Delpech, namely, to avoid suppuration and exfoliation of the tendon, in which he succeeded. Since this last fortunate modification of Delpech's proceeding, several surgeons, among whom I reckon myself, have endeavoured still further to perfect it by suppressing one of the cutaneous punctures. Such has been the development of the *practical* fact. Its essential physiological signification has hitherto remained what it was prior to the curative attempt of the surgeon of Montpellier." (p. 11.)

Before entering on the consideration of the *principle* contended for by M. Guerin, we must remark that we have always considered the great importance attached to the suppression of one of the punctures by the followers of Stromeyer an injustice to that surgeon. The large number of successful cases reported by him show that it is perfectly immaterial as respects cicatrization of the skin and reunion of the tendon whether one or two punctures be made. It should also be borne in mind that by his method of operating a second puncture does not *necessarily* result; he mentions its occurrence as an incidental circumstance only. He endeavours to sever the tendon by pressing the edge of the knife against it, and if the whole is not divided when its point reaches the opposite part of integument he prefers permitting the point to traverse the skin, to disturbing the cellular tissue by sawing or hacking movements of the knife. Unquestionably one puncture is preferable to two, being less painful; and as the section can be effected with equal facility with the point of the blade as with the edge, the operation with a single puncture ought to supersede the other. *The subcutaneous section of a tendon*, whether effected by one or two punctures, remains nevertheless the operation of Stromeyer.

After giving his summary of the successive improvements in the operation of division of tendons, Guerin endeavours to prove that Stromeyer and all succeeding tenotomists have, until his own researches, been entirely ignorant of the reasons of the innocuity of subcutaneous sections. What must Stromeyer, Dieffenbach, and the host of enlightened surgeons who have practised the Stromeyerian operation think of the puerile attempt to deny them the credit of perceiving that the safety of the operation depended on the exclusion of air and absence of inflammation in the severed parts? The immediate closing of the puncture and reunion of the tendon contrasted so strongly with the apprehensions formerly entertained respecting injury of these tissues, that every educated surgeon perceived at once without the two years' reflection it cost M. Guerin to arrive at the same conclusion, (p. 20,) that exclusion of the air and immediate union of the cutaneous section constituted the importance of the Stromeyerian method. M. Guerin imputes (p. 13) to Stromeyer ignorance of "the absence of inflammation and of the occurrence of immediate *organization* so characteristic of subcutaneous tenotomy:" he quotes nevertheless, in corroboration of his assertion, the following passage from Stromeyer's first paper in Rust's Magazin and the Archives de Médecine:* "The object I had in view, to make the external wounds as small as possible, in order to prevent entrance of air, suppuration and exfoliation† of the tendon was so completely effected, that the point of the knife alone penetrated the opposite integument, without occasioning a bleeding wound; the puncture at which the knife entered was not larger than the width of the blade." Stromeyer in this sentence distinctly enounces the concatenation of events to be guarded against, entry of air, suppuration and exfoliation of the tendon. He omits mention of inflammation, one link in the chain of phenomena; but does Stromeyer or any other well-informed surgeon suppose suppuration to ensue without previous inflammation, or that suppuration is not a sign of pre-existing inflammation? Certainly not; and consequently it must be believed that the omission of the word inflammation was accidental. The advantage taken by M. Guerin of this omission (p. 13) is unworthy a candid searcher after truth. Stromeyer does not in any of his works, as Guerin mistates (p. 14), mention that "the reaction was weak, insignificant," implying an acknowledgment of the occurrence of

* Republished in Stromeyer's Beiträge, p. 60.

† It may be here remarked that Guérin's translation of this sentence differs from the above purely literal one, by his having placed the word suppuration after exfoliation which alters the import.—REV.

inflammation in his cases, although in a lessened degree. The only passages on this subject in relation to the subcutaneous section of the tendo Achillis, we can find in Stromeyer's works, are in his Beiträge, pp. 71 and 75. "The small wounds healed *by the first intention.* The *adhesion* of the ends of the tendon was so far advanced," &c. &c. "On the fifth day the ends had adhered"—which prove his opinion to have been, that on subcutaneous section of tendons, reunion occurs not by inflammation and suppuration, but by first intention, by adhesion, or by immediate organization, which term M. Guerin adopts to express this process of union.

M. Guerin, as if in anticipation of his claims being disallowed, is very indignant against the awarders of spurious honours:

"When a discovery is effected, the invention may almost always be attributed to somebody else who does not doubt his share in it. The pretended inventor has only to lend himself to these accidental interpretations, and by means of a few vague expressions, detached words, and ambiguous phrases, he may impose the belief that everything had been observed and described before it was done by him, who really did observe and describe everything........... We repeat for the last time, neither Stromeyer, nor any of those who have adopted his method, have stated that subcutaneous wounds of tendons do not inflame; all have followed Delpech, and preceding writers, and have spoken only of the absence of *suppuration* and exfoliation of tendons, implicitly and explicitly admitting the existence of a certain degree of inflammation. It is therefore evident that in their opinion the essential character of subcutaneous tenotomy is not the production of a distinct order of phenomena, the immediate organization of the divided parts, but the diminution of the inflammatory phenomena of ordinary wounds, that is to say, a feeble reaction, a slight, usually imperceptible suppuration, seldom an abscess: the whole arising from the nature of the tissues divided, and the smallness of the wounds of the skin." (p. 17.)

If the brief quotation we have made from Stromeyer do not enforce conviction that Stromeyer recognized the principle on which the security of his method was founded, we may refer to the opinion of our countryman, Dr. Little, confessedly a close follower in many respects of Stromeyer. He says in his Inaugural Dissertation,* (p. 56,) the first treatise published on the subject in any language, "Thus, usually on the second or third day the small wounds are agglutinated without pain or inflammation." And in his Treatise on Club-foot, &c., reviewed in this Journal, October, 1839, he thus enters more fully into the matter:

"We may explain the reason of the safety of dividing so large a tendon as that of the tendo Achillis, or of puncturing the fascia of the sole of the foot, and dividing the tendon of the flexor longus pollicis, by the facts, that no inflammation follows the infliction of so small a wound, that the skin immediately agglutinates, and the severed tendon is placed in the comparatively safe condition of a ruptured tendon, between which and an exposed wound of tendon there is the same difference as exists between the simple and compound fracture of a bone, with reference to the probabilities of suppuration or sloughing." (Little's Treatise, p. 29.)

M. Guerin, in another part of the memoir (p. 28), avails himself of the same illustration as that above afforded by Dr. Little.

The truly valuable part of M. Guerin's memoir is the generalization of the principle furnished by the mode of healing of subcutaneous section of tendons. M. Guerin lays it down as an axiom "*that all subcutaneous*

* Symbolæ ad Talipedem verum cognoscendum, etc.—*Berolini,* (January,) 1837.

*sections, in whatever situation and whatever be the nature of the structures divided, participate in the property of subcutaneous sections of tendons; that is to say, they do not influence or suppurate, but reunite immediately ;*" and the correctness of this principle is shown by subcutaneous operations on animals, involving in some cases the division of the whole of the muscles, nerves, and vessels, on the posterior aspect of the extremities, and in others by subcutaneous punctures of the articulations, by subcutaneous section of various ligaments in the human subject, and by the subcutaneous puncture of abscesses, subcutaneous section of the tunica-vaginalis for the cure of hydrocele, &c. As further extension of the subcutaneous method of operation proposed by himself or by other surgeons, he mentions:

1. The evacuation of sanguineous tumours formed by subcutaneous effusion of blood and of serous cysts, the occurrence of which he has witnessed after subcutaneous myotomy; restoration being effected by adhesion of the parietes of the cysts without suppuration.
2. The subcutaneous incision of incipient phlegmonous swellings with the view of producing disgorgement.
3. The evacuation of meliceris tumours.
4. The removal of small exostoses.
5. The division of the sphincter ani, for the cure of fissure of the rectum.
6. The subcutaneous section of the stricture of the femoral or inguinal hernia, and adhesion of the neck of the sac.
7. The subcutaneous ligature of veins for the cure of varicocele.
8. The subcutaneous section of the capsular ligament of the knee, for the purpose of exciting effusion of lymph and consequent fixing loose bodies contained in the articulation.
9. The subcutaneous section of nerves, for the cure of neuralgia.

M. Guerin also proposes the evacuation of loose bodies from the knee by a process which has already been often applied by other surgeons with fatal results. It consists in fixing the foreign substance, and forming a large fold of integuments on one side of it; after which the integuments and capsular ligaments require division to an extent sufficient to permit the introduction of forceps to seize the foreign body. The fold of integuments being relaxed, the cutaneous and capsular incisions no longer correspond, and the entry of air is guarded against. We have witnessed the fatal performance of this operation. No necessity existed for the introduction of forceps, as the loose substance slipped out the instant the knife was withdrawn. On the introduction of this operation many years since, it appeared a great improvement; but, in addition to the danger attending it, it is at best very undeserving the title of subcutaneous. Very far different, however, is the elegant, ingenious, and, we conceive, perfectly safe method resorted to by M. Goyrand, of which an account is given in a former Number of this Journal. (vol. XI. p. 526.) Further experience is required to enable us to decide on the general applicability of this operation; but we are much mistaken if M. Goyrand do not earn a lasting reputation in having rendered the removal of foreign bodies from the knee-joint—hitherto considered among the most dangerous operations, and utterly condemned by many of the best surgeons of the present day—one of the simplest, safest, and most effectual.

